# Shifting High-Risk HPV Genotype Patterns in Histologically Confirmed Cervical HSIL: Temporal Changes and Differential Distribution in Invasive Squamous Cell Carcinoma

**DOI:** 10.3390/life16101760

**Published:** 2026-10-21

**Authors:** Cristina Díaz del Arco, Mar Ramírez Mena, Silvia Rojas Calles, Fernando Durán Botía, Mónica Bellón del Amo, Ricardo García Martínez, Luis Ortega Medina

**Affiliations:** 1 Department of Pathology, Health Research Institute of the Hospital Clínico San Carlos (IdISSC), Hospital Clínico San Carlos, 28040 Madrid, Spain; 2 Department of Legal Medicine, Psychiatry and Pathology, School of Medicine, Complutense University of Madrid, 28040 Madrid, Spain; 3 Department of Obstetrics and Gynecology, Health Research Institute of the Hospital Clínico San Carlos (IdISSC), Hospital Clínico San Carlos, 28040 Madrid, Spain; 4 School of Biological Sciences, Complutense University of Madrid, 28040 Madrid, Spain; slvrjs22@gmail.com

**Keywords:** human papillomavirus, HPV genotypes, cervical HSIL, cervical squamous cell carcinoma, cervical cancer, genotype distribution, HPV epidemiology

## Abstract

High-risk human papillomavirus (HR-HPV) genotypes differ in their oncogenic potential and contribution to cervical precancer and invasive cancer. We investigated temporal changes in HR-HPV genotype patterns in histologically confirmed cervical high-grade squamous intraepithelial lesions (HSIL) and differences between 2022–2025 HSIL and invasive squamous cell carcinoma (SCC) in an opportunistic screening setting in Spain. This retrospective study included 371 patients with HSIL from 2011 to 2016, 411 with HSIL from 2022 to 2025, and 65 with SCC from 2022 to 2025. Among HR-HPV-positive HSIL, HPV16/18 frequency decreased from 65.7% to 50.8% (*p* = 0.003), accompanied by a lower mean number of detected genotypes. After adjustment for age and cytological diagnosis, the 2022–2025 period was associated with lower odds of HPV16 (OR 0.59) and HPV18 (OR 0.36) detection. Compared with 2022–2025 HSIL, SCC showed higher HPV16/18 frequency (75.0% vs. 50.8%; *p* = 0.003), fewer multiple infections, and lower representation of other HR-HPV genotypes. Exploratory birth-cohort analysis showed lower HPV16/18 frequency in women born in or after 1994, with higher individual frequencies of HPV52 and HPV58. HR-HPV genotype patterns in histologically confirmed HSIL varied over time and between birth cohorts, while invasive SCC showed greater HPV16/18 representation and lower HPV diversity.

## 1. Introduction

Cervical cancer remains a major health problem despite the availability of effective preventive strategies. In 2022, approximately 662,000 new cases and 349,000 deaths were estimated worldwide, with geographical variation in incidence and mortality [1]. Persistent infection with carcinogenic human papillomavirus (HPV) is the central etiological factor in cervical carcinogenesis, and the development of high-grade squamous intraepithelial lesions (HSIL) represents a critical intermediate step in the pathway leading to invasive squamous cell carcinoma (SCC) [2].

High-risk HPV (HR-HPV) genotypes are not biologically equivalent and have different clinical implications due to their distinct risks of persistence and malignant progression [3,4]. Recent global estimates based on more than 100,000 HPV-positive invasive cervical cancers showed a dominant contribution of HPV16, followed by HPV18, whereas the attributable fractions of other types were lower [3]. Other studies have also shown genotype-specific differences in oncogenic potential [4], supporting genotype-based risk stratification. Extended HPV genotyping is increasingly incorporated into cervical screening and management strategies, as reflected in the 2026 World Health Organization guideline on HPV DNA genotyping [5,6].

HR-HPV genotype distribution differs according to lesion grade [4,7]. Historical comparative data have shown that HPV16, HPV18, and HPV45 are proportionally more frequent in invasive cervical cancer than in high-grade precursor lesions, whereas several other HR-HPV genotypes show the opposite pattern [7]. More recent histology-based studies have also documented this heterogeneity [8,9]. However, the relative frequency of individual genotypes varies geographically [10,11], limiting the direct extrapolation of these findings to different populations. In Spain, histology-based data are limited: a previous study from Madrid included 158 cervical intraepithelial neoplasia grade 2–3 (CIN2–3) lesions but only seven invasive carcinomas diagnosed between 2005 and 2011 [12], whereas a larger study of invasive cervical cancer included cases diagnosed over a long historical period extending from 1940 to 2007 [13]. Updated data are therefore needed to define the genotype profile of HSIL and invasive SCC in the current Spanish setting.

HR-HPV genotype profiles can also change over time in response to epidemiological and ecological factors, including changes in population characteristics, viral circulation, screening practices, and preventive interventions [14,15,16]. Temporal shifts in the relative frequency of individual HPV types have been reported, with trends depending on the population and study period [17,18]. Such variability highlights the value of updated surveillance in different epidemiological contexts.

In a recent study from our institution, we identified temporal changes in HR-HPV prevalence and genotype distribution between two periods of opportunistic cervical screening [19]. However, that study included HPV-tested cervical screening samples regardless of the final diagnosis, with histological evaluation limited to patients who underwent biopsy. In addition, only approximately half of biopsied cases with atypical squamous cells, cannot exclude HSIL (ASC-H) or HSIL cytology showed HSIL or carcinoma on histological examination [19]. Thus, whether the temporal changes in HPV genotype distribution observed in screening samples are also found in histologically confirmed HSIL remains unclear.

The primary aim of the present study was to evaluate temporal changes in HR-HPV genotype distribution in histologically confirmed cervical HSIL diagnosed in 2011–2016 and 2022–2025. As a secondary objective, we compared HR-HPV genotype patterns and infection multiplicity between HSIL and invasive SCC diagnosed in 2022–2025. Finally, an exploratory birth-cohort analysis was performed to assess potential differences in genotype distribution within the 2022–2025 HSIL cohort according to year of birth.

## 2. Materials and Methods

### 2.1. Study Design and Case Selection

This retrospective observational study was conducted at the Department of Pathology of Hospital Clínico San Carlos, a tertiary referral hospital in Madrid, Spain. All patients with a histological diagnosis of HSIL in cervical biopsy specimens processed and archived during two study periods, 2011–2016 (historical cohort) and 2022–2025 (contemporary cohort), were identified. These periods were selected to compare HSIL genotype patterns approximately a decade apart, at different stages following the introduction of systematic HPV vaccination in the Community of Madrid in 2008. Both periods preceded the implementation of organized cervical cancer screening in our area, which began in 2026, allowing the comparison to be conducted within the same opportunistic screening setting.

In addition, all patients diagnosed with SCC during the contemporary period (2022–2025) were included. These cases were used exclusively for a secondary cross-sectional comparison with contemporary HSIL, aimed at characterizing current genotype differences between high-grade precursor lesions and SCC. The primary temporal analysis was restricted to HSIL cases from the two study periods.

All specimens were obtained as part of routine clinical practice. Cases were identified from the institutional pathology information system (AnaPath; Vitro, S.A., Seville, Spain) by searching for cervical biopsies, including incisional and excisional specimens, diagnosed as HSIL or SCC within the defined study periods. Only one case per patient was included, based on the highest-grade histological diagnosis identified during the corresponding study period. When multiple specimens showed the same highest-grade lesion, the earliest specimen was selected.

### 2.2. Histopathological and Cytological Assessment

Biopsy specimens were fixed in 10% neutral-buffered formalin, routinely processed, paraffin-embedded, and stained with hematoxylin and eosin. Histological diagnoses of squamous intraepithelial lesions and invasive SCC were established based on morphological criteria, with intraepithelial lesions classified using the two-tiered terminology of low-grade squamous intraepithelial lesion (LSIL) and HSIL, corresponding to CIN1 and CIN2–3, respectively. In cases with equivocal squamous intraepithelial morphology, particularly when distinguishing LSIL from HSIL on hematoxylin and eosin sections, immunohistochemistry for p16 and Ki-67 was performed at the discretion of the gynecopathologist to support histological classification, following routine laboratory protocols.

Cervical cytology results available before or at the time of histological diagnosis were retrieved from the institutional pathology information system (AnaPath). Cytological specimens were obtained during routine clinical practice in gynecology or primary-care settings and processed using liquid-based cytology (ThinPrep^®^, Hologic, Marlborough, MA, USA). Cytological diagnoses were classified according to the Bethesda System.

### 2.3. HPV Testing and Genotyping

HR-HPV results obtained as part of routine clinical care were retrospectively retrieved when available. HR-HPV testing was performed on residual liquid-based cytology material obtained from the same diagnostic episode as the cervical biopsy or on formalin-fixed, paraffin-embedded (FFPE) tissue from the diagnostic specimen. Cytological material was generally preferred for HPV testing, whereas histological tissue was used when cytology-based testing had not been performed or was not feasible, following routine institutional diagnostic practice. Neither specimen type was designated as the primary analytical source for this retrospective study. For FFPE samples, additional tissue sections were obtained and DNA was extracted using the QIAamp DNA Tissue Kit (Qiagen, Hilden, Germany), following the manufacturer’s instructions.

HR-HPV detection was performed using the cobas^®^ 4800 HPV Test for the 2011–2016 HSIL cohort and the cobas^®^ 5800 HPV Test for the 2022–2025 HSIL and SCC cohorts (Roche Diagnostics, Mannheim, Germany). Both assays belong to the Roche cobas HPV test family and use real-time polymerase chain reaction (PCR) to individually detect HPV16 and HPV18 and collectively detect 12 additional HR-HPV genotypes (HPV31, 33, 35, 39, 45, 51, 52, 56, 58, 59, 66, and 68). The clinical comparability of the two platforms has been demonstrated in an independent validation study [20].

Samples with a positive result for the pooled HR-HPV genotypes underwent extended genotyping using the HPV Direct Flow CHIP (Vitro Master Diagnostica, Seville, Spain), regardless of HPV16 or HPV18 status. The assay is based on PCR amplification of the L1 region followed by reverse dot-blot hybridization and allows identification of individual HR-HPV genotypes and multiple infections. Although the assay detects both high- and low-risk HPV genotypes, only HR-HPV genotypes were analyzed because low-risk HPV testing was not performed in all samples. All molecular assays were performed according to the manufacturers’ instructions and routine laboratory quality-control procedures.

### 2.4. Study Variables and HPV Genotype Classification

Clinical, pathological, and molecular data were retrospectively retrieved from electronic medical records and AnaPath, and entered into an Excel database. Collected variables included age at diagnosis, year of birth, study period, availability and diagnosis of cervical cytology before or at the time of histological diagnosis, histological diagnosis, HR-HPV test availability and result, individual HR-HPV genotype(s), number of detected HR-HPV genotypes, and presence of multiple HR-HPV infection.

For grouped analyses, HR-HPV genotypes were classified into three categories according to vaccine coverage: HPV16/18; other genotypes targeted by the nonavalent HPV vaccine (HPV31/33/45/52/58); and other HR-HPV genotypes not targeted by the nonavalent vaccine (HPV35/39/51/56/59/66/68). These categories were not mutually exclusive in cases with multiple HR-HPV genotypes.

An exploratory birth-cohort analysis was performed within the 2022–2025 HSIL cohort, comparing women born before 1994 with those born in or after 1994. Year of birth was used as an ecological proxy for potential HPV vaccination exposure, based on the introduction of systematic HPV vaccination in the Community of Madrid in 2008, initially targeting girls born in 1994, and the high vaccination coverage achieved in the Community of Madrid according to regional surveillance data [21]. Individual vaccination status was not available; therefore, birth cohort was not considered a measure of individual vaccination status.

### 2.5. Statistical Analysis

Continuous variables are summarized as means and standard deviations (SDs) or medians and ranges, as appropriate, and categorical variables as absolute frequencies and percentages. Continuous variables were compared using Student’s *t*-test or Welch’s *t*-test, as appropriate. Categorical variables were compared using the chi-square test or Fisher’s exact test when expected cell counts were <5. Analyses of individual and grouped genotype distributions, number of detected genotypes, and multiple infection were restricted to HR-HPV-positive cases. HR-HPV-negative cases were retained for overall cohort characterization and comparisons of HPV test results. To assess potential selection bias associated with HPV test availability, patients with and without available HPV test results were compared separately within each HSIL cohort according to age, availability of previous cytology, and cytological diagnosis.

The primary analysis compared HR-HPV genotype patterns between the 2011–2016 and 2022–2025 HSIL cohorts. Multivariable binary logistic regression models were used to assess the association between study period and detection of HPV16 and HPV18, adjusting for age and cytological diagnosis (negative for intraepithelial lesion or malignancy [NILM], atypical squamous cells of undetermined significance [ASC-US]/LSIL, and ASC-H/HSIL). Analyses included cases with complete data for all model covariates. Results are reported as odds ratios (ORs) with 95% confidence intervals (CIs). A secondary analysis compared HR-HPV genotype patterns between the 2022–2025 HSIL and SCC cohorts. Birth-cohort analyses within the 2022–2025 HSIL cohort were considered exploratory. All statistical tests were two-sided, and a *p* value < 0.05 was considered statistically significant. No adjustment for multiple comparisons was applied; therefore, findings from individual genotype comparisons, particularly those arising from secondary and exploratory analyses, should be interpreted with caution. Statistical analyses were performed using IBM SPSS Statistics, version 20.0 (IBM Corp., Armonk, NY, USA).

### 2.6. Ethics

The study was conducted in accordance with the Declaration of Helsinki and applicable legal regulations. The study protocol was approved by the Clinical Research Ethics Committee of Hospital Clínico San Carlos (CEIm-HCSC; internal code 26/140-E; approval record 2.1/26) on 11 February 2026. The requirement for written informed consent was waived by the Ethics Committee. Procedures for the handling of personal data were approved as part of the study protocol.

## 3. Results

### 3.1. Study Cohort Characteristics

**Figure 1:**
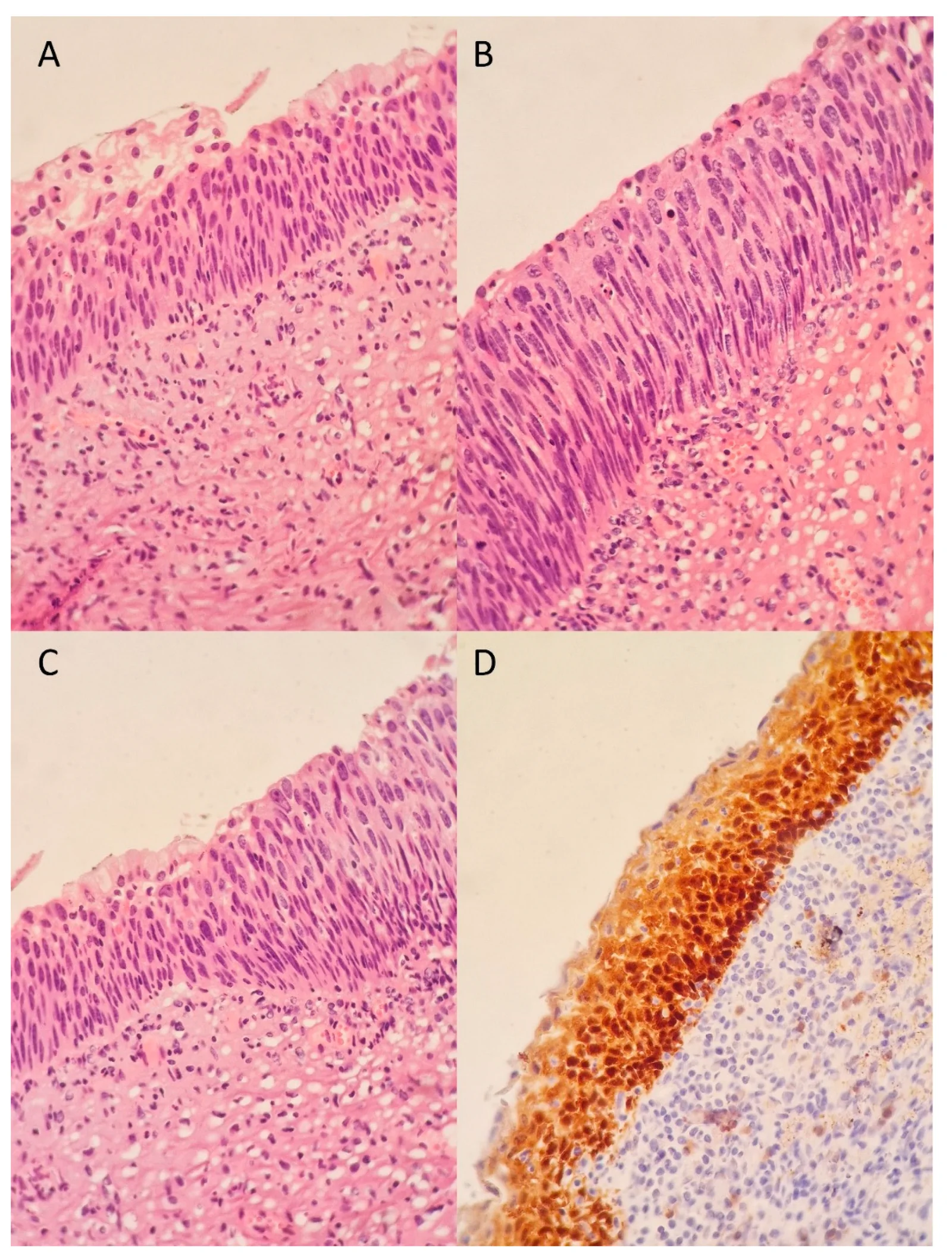
Representative histopathological and immunohistochemical features of cervical high-grade squamous intraepithelial lesions (HSIL). (**A**–**C**) Hematoxylin and eosin staining showing full-thickness loss of epithelial maturation with cytological atypia and mitotic figures extending into the upper epithelial layers (×400). (**D**) Diffuse block-type p16 overexpression (×400).

**Figure 2:**
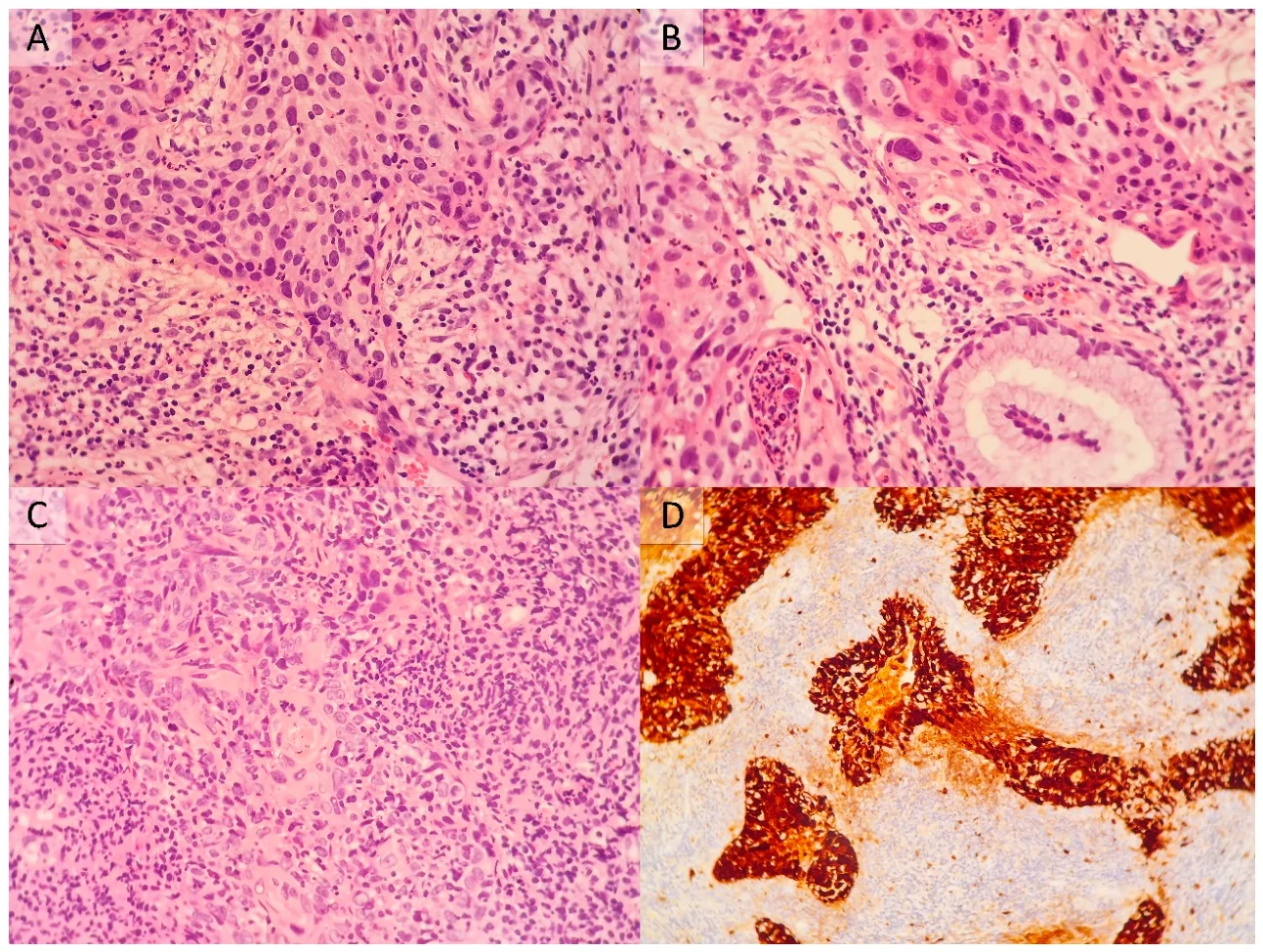
Representative histopathological and immunohistochemical features of invasive cervical squamous cell carcinomas. (**A**–**C**) Hematoxylin and eosin staining showing irregular nests of invasive squamous carcinoma infiltrating the cervical stroma, with focal keratinization (×400). (**D**) Diffuse p16 overexpression in tumor cells (×200).

The study included 847 patients: 371 with histologically confirmed HSIL diagnosed in 2011–2016, 411 with HSIL diagnosed in 2022–2025, and 65 with invasive SCC diagnosed in 2022–2025. Mean age at histological diagnosis was 38 years (SD 10), 41 years (SD 11), and 53 years (SD 16), respectively. The corresponding median ages were 36 (range, 20–77), 40 (range, 21–76), and 51 years (range, 28–94). The clinicopathological characteristics of the study cohorts are summarized in Table 1. Representative histopathological and immunohistochemical findings of HSIL and invasive SCC are shown in Figure 1 and Figure 2.

Cytological diagnosis was available in 84.1% of patients in the 2011–2016 HSIL cohort, 99.3% in the 2022–2025 HSIL cohort, and 50.8% in the SCC cohort. ASC-H/HSIL accounted for 58.9% of cytological diagnoses in the 2011–2016 HSIL cohort and 66.7% in the 2022–2025 HSIL cohort, whereas ASC-H, HSIL, or cytology suspicious for SCC accounted for 87.1% in the SCC cohort.

HR-HPV testing was available in 43.1% of patients in the 2011–2016 HSIL cohort, 83.2% in the 2022–2025 HSIL cohort, and 80.0% in the SCC cohort. Of those with available HPV testing, HR-HPV was detected in 89.4%, 89.2%, and 84.6% of cases, respectively.

To assess potential selection bias associated with HPV test availability, clinicopathological characteristics were compared between HSIL patients with and without available HPV results within each study period. In the 2011–2016 cohort, mean age was similar between patients with and without HPV results (39.02 vs. 38.52 years; *p* = 0.656). In the 2022–2025 cohort, patients with available HPV results were slightly older (42.21 vs. 38.57 years; *p* = 0.005), although the effect size was small (Cohen’s d = 0.33). No significant differences in the distribution across five age categories were observed in either cohort (*p* = 0.640 and *p* = 0.280, respectively). Previous cytology was more frequently available among HPV-tested patients in the historical cohort (90.6% vs. 79.1%; *p* = 0.003), whereas availability was similarly high in both groups in the contemporary cohort (99.4% vs. 98.6%; Fisher’s exact *p* = 0.425). The distribution of previous cytological diagnoses differed according to HPV test availability in both cohorts (*p* < 0.001 and *p* = 0.011, respectively), with a higher proportion of ASC-H/HSIL cytology among patients without available HPV results (70.5% vs. 45.5% in 2011–2016 and 82.4% vs. 63.6% in 2022–2025).

Subsequent analyses of HR-HPV genotype distribution, number of detected genotypes, and multiple HR-HPV infection were restricted to HR-HPV-positive cases (*n* = 143, *n* = 305, and *n* = 44, respectively).

### 3.2. Temporal Changes in HR-HPV Genotype Patterns in HSIL (2011–2016 vs. 2022–2025)

Among HR-HPV-positive HSIL cases, the mean number of detected genotypes decreased from 1.6 (SD 0.9) in the 2011–2016 cohort to 1.4 (SD 0.6) in the 2022–2025 cohort (*p* = 0.010). Multiple HR-HPV infections were identified in 35.0% and 28.2% of cases, respectively (*p* = 0.146).

HPV16 was the most frequently detected genotype in both periods, although its frequency decreased from 58.7% in 2011–2016 to 47.2% in 2022–2025 (*p* = 0.023). HPV18 also decreased, from 11.2% to 4.9% (*p* = 0.015). No statistically significant differences were observed for the remaining individual HR-HPV genotypes (Table S1).

In the grouped-genotype analysis, HPV16/18 were detected in 65.7% of cases in 2011–2016 and 50.8% in 2022–2025 (*p* = 0.003). No significant temporal differences were observed for the other genotype groups (Table S1).

In multivariable logistic regression analysis, diagnosis in 2022–2025 was associated with lower odds of HPV16 detection compared with 2011–2016 (OR 0.59, 95% CI 0.38–0.91; *p* = 0.016), after adjustment for age and cytological diagnosis. Neither age nor cytological diagnosis was independently associated with HPV16 detection. Similarly, diagnosis in 2022–2025 was associated with lower odds of HPV18 detection (OR 0.36, 95% CI 0.17–0.78; *p* = 0.009), whereas age and cytological diagnosis were not statistically significant in the adjusted model (Table 2).

### 3.3. Comparison of 2022–2025 HSIL and Invasive SCC

Among HR-HPV-positive cases diagnosed in 2022–2025, SCC showed a lower mean number of detected HR-HPV genotypes than HSIL (1.1 [SD 0.4] vs. 1.4 [SD 0.6]; *p* = 0.001). Multiple HR-HPV infections were also less frequent in SCC than in HSIL (9.1% vs. 28.2%; *p* = 0.007).

HPV16 was detected more frequently in SCC than in HSIL (65.9% vs. 47.2%; *p* = 0.020). In contrast, HPV31 (4.5% vs. 17.0%; *p* = 0.032), HPV52 (0% vs. 11.1%; *p* = 0.013), and HPV58 (0% vs. 8.9%; *p* = 0.034) were less frequently detected in SCC. No statistically significant differences were observed for the remaining individual HR-HPV genotypes (Table S2).

**Figure 3:**
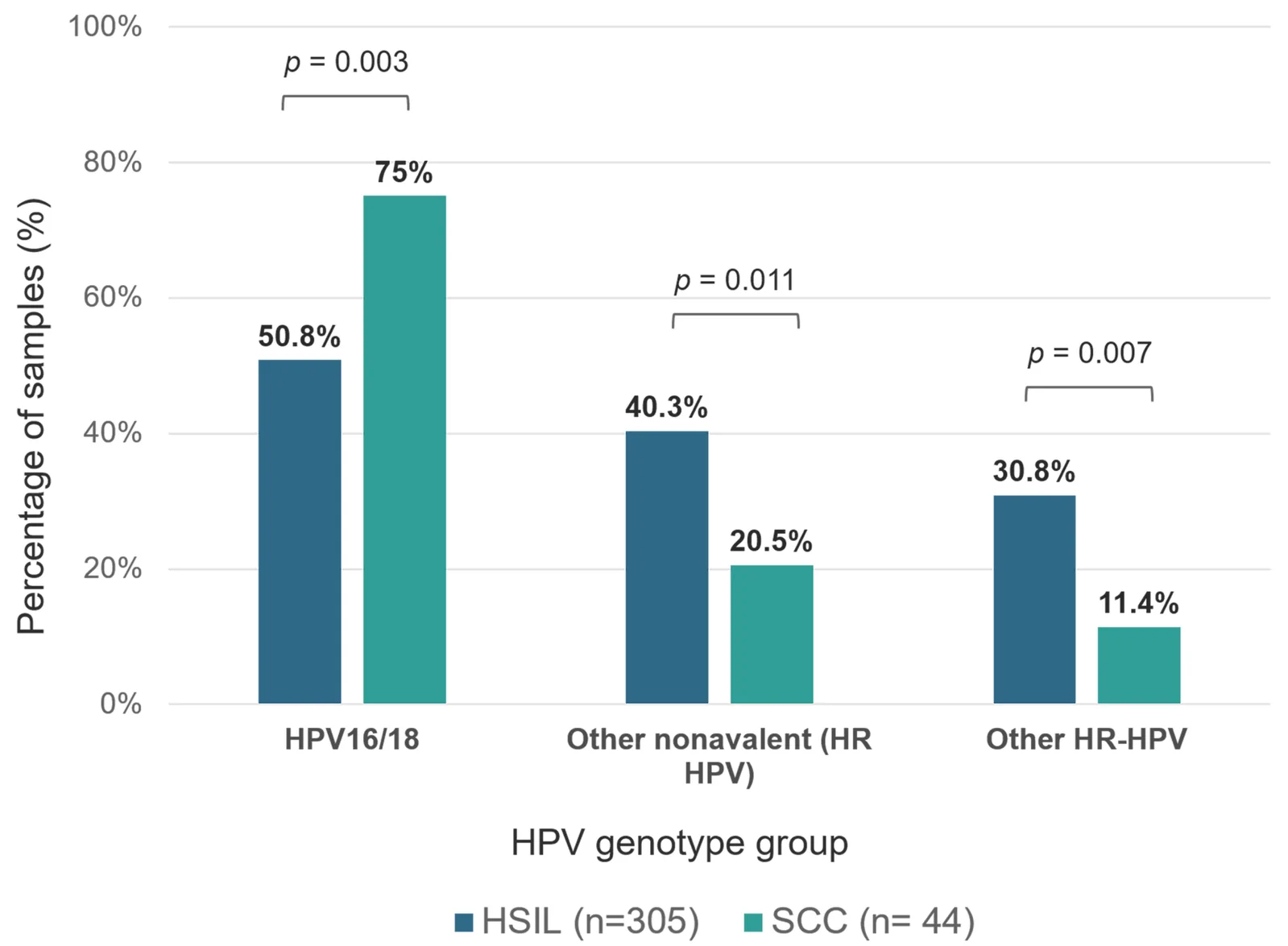
Grouped HR-HPV genotype distribution in 2022–2025 HSIL and invasive SCC. Frequencies of HPV16/18, other nonavalent vaccine genotypes (HPV31/33/45/52/58), and other HR-HPV genotypes (HPV35/39/51/56/59/66/68) among HR-HPV-positive HSIL (n = 305) and SCC (n = 44) diagnosed in 2022–2025 are shown. Grouped genotype categories are not mutually exclusive because patients with multiple HR-HPV infections may contribute to more than one category. *p* values refer to comparisons between HSIL and SCC for each genotype group. Abbreviations: HPV, human papillomavirus; HR-HPV, high-risk human papillomavirus; HSIL, high-grade squamous intraepithelial lesion; SCC, squamous cell carcinoma.

In the grouped-genotype analysis, HPV16/18 were detected in 75.0% of SCC cases compared with 50.8% of HSIL cases (*p* = 0.003). Conversely, other nonavalent vaccine genotypes (HPV31/33/45/52/58) were detected less frequently in SCC than in HSIL (20.5% vs. 40.3%; *p* = 0.011), as were the remaining HR-HPV genotypes (11.4% vs. 30.8%; *p* = 0.007) (Figure 3).

### 3.4. Birth-Cohort Analysis in 2022–2025 HSIL

**Figure 4:**
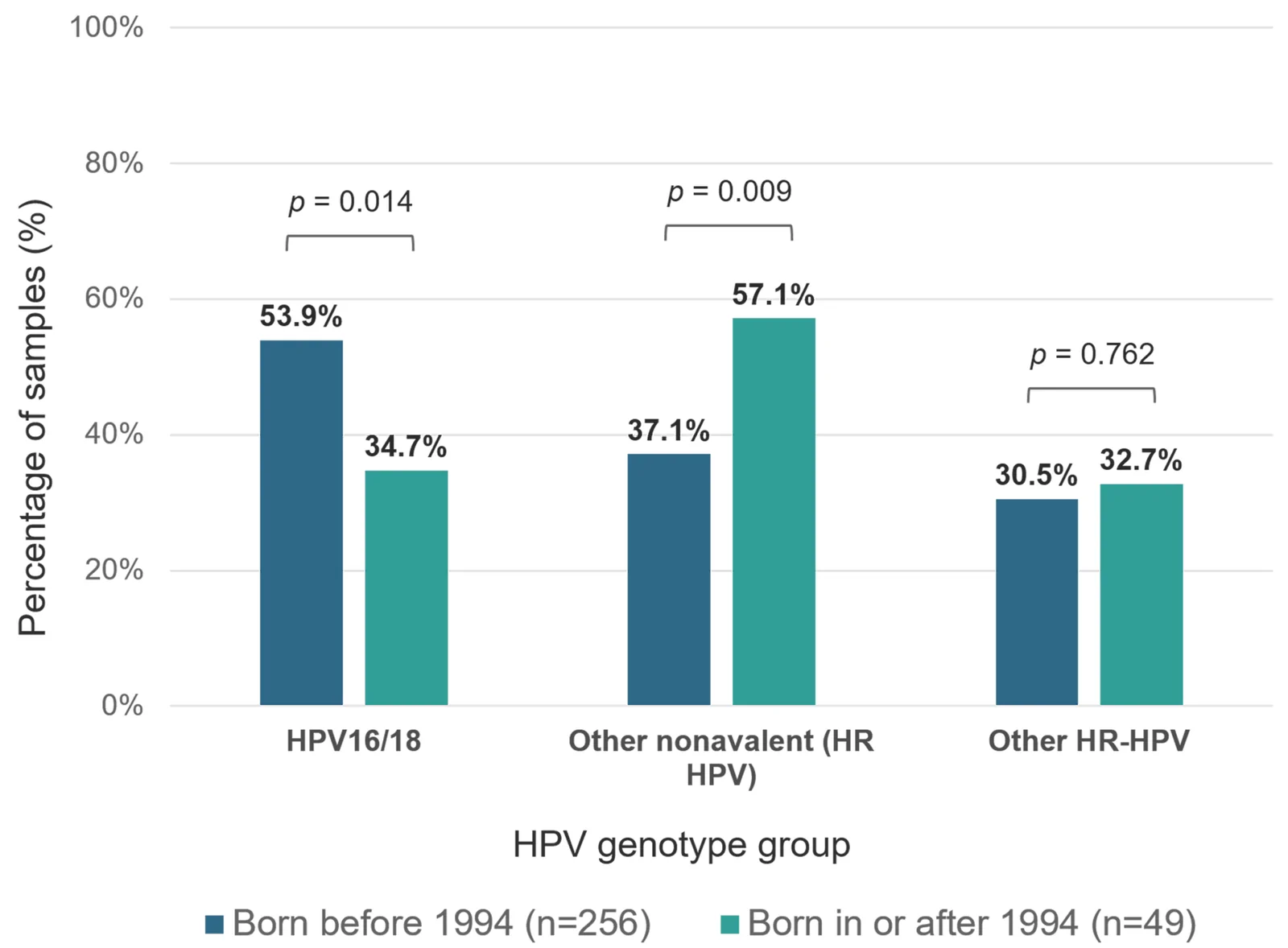
Grouped HR-HPV genotype distribution in 2022–2025 HSIL according to birth cohort. Frequencies of HPV16/18, other nonavalent vaccine genotypes (HPV31/33/45/52/58), and other HR-HPV genotypes (HPV35/39/51/56/59/66/68) among HR-HPV-positive HSIL (n = 305) and SCC (n = 44) diagnosed in 2022–2025 are shown. Grouped genotype categories are not mutually exclusive because patients with multiple HR-HPV infections may contribute to more than one category. *p* values refer to comparisons between HSIL and SCC for each genotype group. Abbreviations: HPV, human papillomavirus; HR-HPV, high-risk human papillomavirus; HSIL, high-grade squamous intraepithelial lesion; SCC, squamous cell carcinoma.

Among the 305 HR-HPV-positive HSIL cases diagnosed in 2022–2025, 256 occurred in women born before 1994 and 49 in women born in or after 1994. In the grouped-genotype analysis, HPV16/18 were less frequent in women born in or after 1994 than in those born before 1994 (34.7% vs. 53.9%; *p* = 0.014). Conversely, other nonavalent vaccine genotypes (HPV31/33/45/52/58) were more frequent in the younger birth cohort (57.1% vs. 37.1%; *p* = 0.009), whereas no difference was observed for the remaining HR-HPV genotypes (32.7% vs. 30.5%; *p* = 0.762) (Table S3 and Figure 4).

At the individual genotype level, HPV16 was detected less frequently in women born in or after 1994 than in those born before 1994 (34.7% vs. 49.6%; *p* = 0.055), although the difference did not reach statistical significance. Significant differences were observed for HPV52 (22.4% vs. 9.0%; *p* = 0.006) and HPV58 (22.4% vs. 6.3%; *p* = 0.001), both of which were more frequent in the younger birth cohort. No significant differences were observed for the remaining individual HR-HPV genotypes (Table S3).

### 3.5. Genotype-Specific Coinfection Patterns

Genotype-specific coinfection patterns were further explored among HR-HPV-positive cases. HPV16 was predominantly detected as a single infection in all study groups. Coinfection among HPV16-positive cases decreased from 35.7% in 2011–2016 HSIL to 25.7% in 2022–2025 HSIL and was lowest in 2022–2025 SCC (10.3%). In contrast, many other HR-HPV genotypes were more frequently detected as part of multiple infections in HSIL, although frequencies varied considerably by genotype. Because several genotypes were represented by small numbers of cases, these findings were considered descriptive. Complete genotype-specific coinfection data are provided in Table S4.

## 4. Discussion

In this retrospective study, we identified temporal changes in HR-HPV genotype distribution in histologically confirmed HSIL and differences between contemporary HSIL and invasive SCC. HPV16/18 frequency decreased in HSIL between 2011–2016 and 2022–2025, with lower odds of both HPV16 and HPV18 detection in the latter period after adjustment for age and cytological diagnosis. In contrast, SCC diagnosed in 2022–2025 showed a higher frequency of HPV16/18 than HSIL from the same period, with fewer detected genotypes and less frequent multiple HR-HPV infection. Exploratory analysis also identified birth-cohort differences, with lower HPV16/18 and higher HPV52 and HPV58 frequencies in women born in or after 1994. Overall, these findings indicate that HR-HPV genotype patterns differ over time, between birth cohorts, and between high-grade precursor and invasive squamous disease.

### 4.1. Temporal Changes in HR-HPV Genotype Patterns in HSIL

The main temporal finding was the reduction in HPV16/18 among histologically confirmed HSIL, from 65.7% in 2011–2016 to 50.8% in 2022–2025. This reflected decreases in both HPV16 and HPV18, and both associations remained significant after adjustment for age and cytological diagnosis. In our previous study of opportunistic cervical screening samples from the same institution, HR-HPV prevalence, genotype distribution, and coinfection patterns also changed between historical and contemporary periods [19]. The present study extends that observation to biopsy-proven high-grade disease, a clinically relevant precursor of invasive cervical cancer.

Temporal changes in genotype composition have been reported in histology-based studies of high-grade lesions in other settings. In a multicenter Italian study of women younger than 30 years with CIN3 diagnosed between 2007 and 2018, Giannella et al. described a decline in HPV16/18 prevalence from 77.8% to 66.0% [22]. Although only unvaccinated women were included, the authors considered herd protection as a possible contributor. Similarly, a longitudinal study of HPV-positive histological samples collected in France between 2005 and 2021 documented temporal fluctuations in individual HPV genotypes, including HPV52 [23]. Evidence from vaccinated populations further supports a role for vaccination in shaping genotype composition: studies from Scotland, Denmark, and Italy have reported reductions in HPV16/18-related infection or high-grade disease in vaccinated women, accompanied by a greater relative contribution of other genotypes [24,25,26]. Together, these studies indicate that the genotype profile of high-grade cervical lesions can vary over time in response to epidemiological trends and preventive interventions.

However, similar trends have not been observed in all settings. A recent French multicenter study of histologically confirmed CIN2/3 lesions found no significant association between birth cohort and HPV16 or HPV18 prevalence in a population with historically low vaccination coverage [27]. This heterogeneity reinforces the value of local lesion-based surveillance and suggests that the factors underlying temporal shifts may differ between populations. Our study could not determine the relative contribution of these factors, as individual vaccination status and information on other potential determinants, such as sexual behavior, were unavailable. Given the historically high HPV vaccination coverage in the Community of Madrid, vaccination may have contributed to the observed reduction in HPV16/18, but its specific contribution cannot be established from our data. Indirect protection through herd immunity, changes in HPV circulation, and differences in the characteristics of the populations undergoing screening may also have influenced the observed temporal patterns.

### 4.2. HR-HPV Genotype Distribution in 2022–2025 HSIL and Invasive SCC

In our study, contemporary HSIL and SCC showed contrasting HR-HPV genotype patterns. At the individual genotype level, HPV16 was more frequent in SCC, whereas HPV31, HPV52, and HPV58 were less frequent. In the grouped analysis, HPV16/18 were detected in 75.0% of HR-HPV-positive SCC compared with 50.8% of HSIL, while both other nonavalent vaccine genotypes and the remaining HR-HPV genotypes were less represented in SCC. However, given the limited number of HR-HPV-positive SCC cases (*n* = 44), differences involving individual genotypes, particularly those detected infrequently, should be interpreted cautiously.

Comparative studies have also found differences in HPV genotype distribution between high-grade precursor lesions and invasive cervical cancer. In a European analysis of high-grade CIN and invasive cervical cancer, HPV16 was the most frequent genotype in both groups. HPV16, HPV18, and HPV45 were 1.1-, 3.5-, and 2.5-fold more frequent in invasive cancer than in high-grade CIN, respectively [28]. The enrichment of HPV18 and HPV45 was particularly evident in adenocarcinomas, whereas HPV16 remained the dominant genotype in squamous carcinoma. These findings complement the historical meta-analysis by Clifford et al. [7] and illustrate that the relative frequency of individual genotypes differs between HSIL and SCC.

Recent histology-based studies have described a similar genotype-specific pattern with increasing disease severity. In a Chinese multicenter study of HR-HPV-positive women referred to colposcopy, HPV16 increased with lesion severity, whereas HPV52 and HPV58 decreased [29]. Another tissue-based series from China found HPV16 to be the most common genotype in both CIN2/3 and SCC, while HPV52 and HPV58 ranked second and third in CIN2/3 and, despite their lower prevalence, remained frequent in SCC [30]. More recently, Nakamura et al. reported HPV16 frequencies of 33.2% in CIN2, 50.1% in CIN3, and 61.7% in SCC, with lower representation of HPV52 and HPV58 in SCC [9]. A comprehensive review by Ye et al. similarly identified HPV16 as the predominant genotype in both CIN2/3 and SCC, with greater representation in invasive carcinoma and geographical variability in the distribution of other HR-HPV genotypes [31]. Thus, the higher frequency of HPV16 and the lower frequencies of HPV31, HPV52, and HPV58 observed in our SCC cohort are consistent with the general trend described with increasing severity of squamous disease, while the relative contribution of non-HPV16 types may vary geographically.

In Spain, a previous histology-based study from Madrid found HPV16/18 in 61.4% of 158 CIN2–3 lesions and 85.7% of seven invasive cervical cancers [12]. A larger Spanish study of 1043 invasive cervical cancers found that HPV16 and HPV18 together accounted for 72.4% of HPV-positive tumors [13]. However, recent Spanish histology-based studies directly comparing HSIL and invasive SCC are scarce.

Although our cross-sectional comparison cannot assess progression from HSIL to SCC, the differences in genotype prevalence observed between these lesions can be interpreted in the context of previous evidence on genotype-specific oncogenic potential. Prospective studies have shown differences among HR-HPV genotypes in persistence and risk of CIN2+ and more advanced disease [4], while global attribution analyses show that HPV16 accounts for a disproportionate share of invasive cervical cancers [3]. Such heterogeneity is biologically plausible: although HR-HPV genotypes share key E6- and E7-mediated transforming mechanisms, experimental studies have shown genotype-specific differences in the activity of these oncoproteins and in their capacity to immortalize human keratinocytes [32,33]. These findings should nevertheless be interpreted at the population level and do not imply that an HPV16-positive HSIL will necessarily progress, or that lesions associated with HPV31, HPV52, or HPV58 will not. Because HSIL and SCC in our study were diagnosed in different patients, the cross-sectional comparison cannot establish genotype-specific progression.

### 4.3. Infection Multiplicity and Genotype-Specific Coinfection Patterns

Patterns of infection multiplicity varied over time and between HSIL and SCC. Between the two HSIL periods, the mean number of detected HR-HPV genotypes decreased from 1.6 to 1.4, although the reduction in multiple infections from 35.0% to 28.2% was not statistically significant. In 2022–2025, SCC showed significantly fewer multiple HR-HPV infections than HSIL, with a lower mean number of detected genotypes. Among HPV16-positive cases, additional HR-HPV genotypes were detected in 25.7% of 2022–2025 HSIL and only 10.3% of SCC.

Several studies have reported fewer multiple infections with increasing lesion severity. In a study of women with prior HPV genotyping and histopathological follow-up, single HR-HPV infection became more frequent as lesion severity increased, whereas multiple infections decreased [34]. Koç et al. reported multiple-type infections in 31.4% of LSIL, 19.0% of HSIL, and 12.5% of cervical cancers in a histology-based series [35]. Using next-generation sequencing, Basiletti et al. also found multiple infections more often in ≤CIN1 than in CIN3+ (85% vs. 41%) [36]. However, this gradient has not been consistently observed: a multicenter Chinese study including women with histologically diagnosed CIN or invasive cervical cancer found no significant difference in multiple-type infection frequency across lesion categories [37].

The biological significance of multiple HR-HPV infection remains uncertain. A retrospective multicenter study found that multiple infection was associated with higher odds of CIN1 (OR 1.44) and CIN2 (OR 1.70) compared with single infection, but not CIN3 (OR 1.08, 95% CI 0.86–1.37) [38]. Other studies suggest that risk may depend more on the genotype present than on the number of detected genotypes. In a cervical screening study, multiple infection did not increase the risk of CIN2+ when HPV16 was present, and the genotype with the highest risk appeared to account for most of the risk within a coinfection [39]. Song et al. similarly found no significant increase in risk with coinfection for most HR-HPV genotypes, although associations differed for some individual types, including HPV51 [40]. Likewise, a 2026 study including histological diagnoses ranging from normal cervix to SCC found no higher risk of CIN2+ for HPV16-containing multiple infections than for HPV16 single infection [41].

Taken together, our results are compatible with lower detectable HPV diversity in invasive SCC and a greater representation of selected oncogenic genotypes, particularly HPV16. However, because our comparison is cross-sectional, we cannot determine why multiple infections are less frequent in SCC than in HSIL. Possible explanations include genotype-specific differences in persistence, loss of accompanying infections, and differences in carcinogenic potential among the genotypes detected in precursor and invasive lesions.

### 4.4. Birth-Cohort Differences in 2022–2025 HSIL

The exploratory birth-cohort analysis identified variation in genotype composition within 2022–2025 HSIL. Women born in or after 1994 had a lower frequency of HPV16/18 than women born before 1994, whereas other nonavalent vaccine genotypes were more frequent. At the individual genotype level, HPV52 and HPV58 were significantly more common in the younger birth cohort, while no significant differences were observed for the remaining HR-HPV genotypes.

As discussed above, studies from vaccinated populations have shown reductions in HPV16/18-related high-grade lesions, with consequent changes in the relative genotype composition of the lesions that remain [24,25,26]. This pattern is consistent with the genotype-specific protection provided by prophylactic HPV vaccines [42,43].

Nevertheless, our birth-cohort findings should be interpreted cautiously. Individual vaccination status was not available, and birth in or after 1994 was used as an ecological proxy for potential vaccine exposure. Furthermore, the younger group included only 49 HR-HPV-positive HSIL cases. As previously noted, no significant birth-cohort differences in HPV16/18 prevalence were observed in the French CIN2/3 study [27]. In contrast, a multicenter study of 1332 unvaccinated women with CIN3 by Giannella et al. identified age-related variation in genotype distribution, with the proportion of lesions associated with non-HPV16/18 genotypes increasing with age [44]. Although these studies examined different populations and genotype groupings, they suggest that age-related differences in HPV genotype distribution are neither universal nor necessarily dependent on vaccination. In our study, the observed birth-cohort differences could be related to direct or indirect effects of HPV vaccination, but they might also reflect other epidemiological factors, including generational differences in sexual behavior and HPV exposure, as well as age-related differences in viral persistence or reactivation. The relative contribution of these factors cannot be established from our data.

The higher frequencies of HPV52 and HPV58 in younger women should also not be interpreted as evidence of genotype replacement, because a reduction in HPV16/18-associated lesions may increase the relative contribution of other genotypes without an increase in their absolute frequency. Our findings indicate a shift in the genotype composition of HSIL in the younger birth cohort, rather than demonstrating an increase in the incidence of HPV52- or HPV58-associated lesions. Whether this pattern reflects redistribution among genotypes or changes in genotype-specific incidence will require further population-based evaluation.

### 4.5. Implications

Our findings support the value of genotype-specific surveillance in histologically confirmed HSIL, as this represents a clinically relevant precursor lesion in cervical carcinogenesis. The temporal and birth-cohort differences observed in our HSIL cohorts indicate that the relative contribution of HR-HPV genotypes to high-grade disease is not static. In addition, the higher frequency of HPV16/18 and lower genotype diversity in SCC show that genotype patterns in HSIL do not directly mirror those observed in invasive disease.

As vaccination coverage increases and extended genotyping becomes more common in cervical screening, these observations support monitoring genotype-specific data in both histologically confirmed precancer and invasive cancer. Such surveillance could distinguish changes in overall HR-HPV prevalence from shifts in the genotypes associated with clinically relevant disease and assess how these patterns evolve as prevention strategies change.

### 4.6. Strengths and Limitations

The main strengths of this study include the use of histologically confirmed cervical lesions, the inclusion of two well-defined HSIL cohorts separated by approximately a decade, and the concurrent evaluation of HSIL and invasive SCC during the contemporary period. Restriction to one diagnostic biopsy per patient avoided repeated observations from the same individual, while extended genotyping allowed assessment of individual HR-HPV genotypes, vaccine-related genotype groups, and infection multiplicity. The relatively large number of biopsy-confirmed HSIL cases also enabled adjustment of the main temporal associations for age and cytological diagnosis. Finally, the use of cases from the same institution and the same opportunistic screening setting provided a consistent clinical context, while standardized sample handling and laboratory procedures ensured methodological consistency across study periods.

Several limitations should also be considered. First, the retrospective, single-center design may limit the generalizability of the findings to other populations and screening settings. Second, HR-HPV testing was not available for all patients and was substantially less frequent among HSIL cases in 2011–2016 than in 2022–2025 (43.1% vs. 83.2%). Additional comparisons according to HPV test availability showed no significant differences in age distribution across categories within either HSIL cohort, although a small difference in mean age was observed in the contemporary cohort. Differences in cytological findings were observed in both periods, potentially reflecting clinical testing pathways associated with previous cytological abnormalities. Although the clinical criteria for requesting HPV testing did not formally change between study periods, the reasons underlying this difference in test availability could not be determined retrospectively. The main temporal analyses were adjusted for age and cytological diagnosis; nevertheless, residual selection bias cannot be completely excluded. Third, individual HPV vaccination status was unavailable. Consequently, the birth-cohort analysis should be regarded as exploratory, with year of birth representing only an ecological proxy for potential vaccination exposure; the observed genotype differences cannot be attributed directly to vaccination. Fourth, the number of HR-HPV-positive SCC cases was relatively small (*n* = 44), limiting the precision of comparisons involving less prevalent genotypes. In addition, because no adjustment for multiple comparisons was applied, some statistically significant differences in individual genotype frequencies may represent chance findings, particularly in secondary and exploratory analyses. Finally, the comparison between contemporary HSIL and SCC was cross-sectional and cannot establish genotype-specific progression from HSIL to invasive carcinoma.

## 5. Conclusions

This study identifies temporal changes in HR-HPV genotype distribution among histologically confirmed cervical HSIL, characterized by a lower representation of HPV16/18 in 2022–2025 than in 2011–2016. In contrast, invasive SCC diagnosed in 2022–2025 showed a higher representation of HPV16/18, fewer detected HR-HPV genotypes, and a lower frequency of multiple infections than HSIL. The exploratory birth-cohort analysis further showed lower HPV16/18 and higher HPV52/58 frequencies among women born in or after 1994. Overall, these findings indicate that HR-HPV genotype patterns vary over time, between birth cohorts, and between high-grade precursor and invasive squamous lesions, supporting continued genotype-specific surveillance of both cervical precancer and cancer.

## 6. Key Findings

• The proportion of HR-HPV-positive HSIL associated with HPV16/18 decreased from 65.7% in 2011–2016 to 50.8% in 2022–2025, and the temporal association remained significant for both HPV16 and HPV18 after adjustment for age and cytological diagnosis.
• In 2022–2025, invasive SCC showed a higher frequency of HPV16/18 than HSIL (75.0% vs. 50.8%; *p* = 0.003), together with a lower number of detected HR-HPV genotypes and a lower frequency of multiple infections.
• Among 2022–2025 HSIL, women born in or after 1994 showed a significantly lower frequency of HPV16/18 and significantly higher frequencies of HPV52 and HPV58 than women born before 1994.
• Overall, the findings indicate temporal and birth-cohort differences in HR-HPV genotype patterns in cervical HSIL, together with greater representation of HPV16/18 in invasive SCC.

## Figures and Tables

**Table 1:** Clinicopathological characteristics of the study cohorts.

Characteristic	HSIL 2011–2016 (*n* = 371)	HSIL 2022–2025 (*n* = 411)	SCC 2022–2025 (*n* = 65)
Age, years, mean (SD)	38 (10)	41 (11)	53 (16)
Age, years, median (range)	36 (20–77)	40 (21–76)	51 (28–94)
Born in or after 1994, *n* (%)	1/368 (0.3)	68/411 (16.5)	3/65 (4.6)
Cervical cytology available, *n* (%)	312/371 (84.1)	408/411 (99.3)	33/65 (50.8)
**Cytological diagnosis, *n*/*N* (%)**			
NILM	21/309 (6.8)	48/406 (11.8)	2/31 (6.5)
ASC-US	27/309 (8.7)	26/406 (6.4)	2/31 (6.5)
LSIL	79/309 (25.6)	61/406 (15.0)	0/31 (0)
ASC-H	34/309 (11.0)	89/406 (21.9)	7/31 (22.6)
HSIL	148/309 (47.9)	182/406 (44.8)	17/31 (54.8)
Suspicious for SCC	0/309 (0)	0/406 (0)	3/31 (9.7)
HR-HPV testing available, *n* (%)	160/371 (43.1)	342/411 (83.2)	52/65 (80.0)
HR-HPV positive, *n*/*N* (%)	143/160 (89.4)	305/342 (89.2)	44/52 (84.6)

Data are presented as *n*/*N* (%) unless otherwise indicated. Percentages are based on available data for each variable, and denominators may vary because of missing data. HR-HPV positivity was calculated for cases with available HPV test results. Abbreviations: ASC-H, atypical squamous cells, cannot exclude HSIL; ASC-US, atypical squamous cells of undetermined significance; HR-HPV, high-risk human papillomavirus; HSIL, high-grade squamous intraepithelial lesion; LSIL, low-grade squamous intraepithelial lesion; NILM, negative for intraepithelial lesion or malignancy; SCC, squamous cell carcinoma; SD, standard deviation.

**Table 2:** Multivariable logistic regression analyses of HPV16 and HPV18 detection among HR-HPV-positive HSIL cases.

Variable	Adjusted OR	95% CI	*p* Value
**HPV16**			
Study period, 2022–2025 vs. 2011–2016	0.59	0.38–0.91	0.016
Age, per year	1.01	0.99–1.03	0.166
Cytological diagnosis			0.821
ASC-US/LSIL vs. NILM	0.96	0.49–1.91	0.912
ASC-H/HSIL vs. NILM	1.10	0.58–2.09	0.769
**HPV18**			
Study period, 2022–2025 vs. 2011–2016	0.36	0.17–0.78	0.009
Age, per year	1.03	1.00–1.06	0.079
Cytological diagnosis			0.303
ASC-US/LSIL vs. NILM	0.99	0.30–3.25	0.988
ASC-H/HSIL vs. NILM	0.55	0.17–1.79	0.317

Separate binary logistic regression models for HPV16 and HPV18 detection included 429 HR-HPV-positive HSIL cases with complete data for study period, age at diagnosis, and cytological diagnosis. Reference categories were 2011–2016 for study period and NILM for cytological diagnosis. Adjusted ORs for age correspond to a one-year increase. Abbreviations: ASC-H, atypical squamous cells, cannot exclude HSIL; ASC-US, atypical squamous cells of undetermined significance; CI, confidence interval; HPV, human papillomavirus; HSIL, high-grade squamous intraepithelial lesion; LSIL, low-grade squamous intraepithelial lesion; NILM, negative for intraepithelial lesion or malignancy; OR, odds ratio.

## Data Availability

The data supporting the findings of this study are available from the corresponding author upon reasonable request.

## Supplementary Materials

The following supporting information can be downloaded at: https://www.mdpi.com/article/10.3390/life16101760/s1. Table S1: HR-HPV genotype distribution in HSIL according to study period; Table S2: HR-HPV genotype distribution in 2022–2025 HSIL and invasive SCC; Table S3: HR-HPV genotype distribution in 2022–2025 HSIL according to birth cohort; Table S4: Genotype-specific frequency of multiple HR-HPV infection according to study cohort.
