# Supplementary material for: Shifting High-Risk HPV Genotype Patterns in Histologically Confirmed Cervical HSIL: Temporal Changes and Differential Distribution in Invasive Squamous Cell Carcinoma
Source: Life (Basel). 2026 Oct 21;16(10):1760. doi: 10.3390/life16101760 (PMC13705950; doi:10.3390/life16101760)
Supplement: Supplementary file 1 [file life-16-01760-s001.zip › life-4591808-supplementary.pdf]

SUPPLEMENTARY MATERIAL

Table S1. HR-HPV genotype distribution in HSIL according to study period

**Variable**	**HSIL 2011–2016 (n = 143)**	**HSIL 2022–2025 (n = 305)**	***p* value**
Number of HR-HPV genotypes, mean (SD)	1.6 (0.9)	1.4 (0.6)	0.010
Multiple HR-HPV infection, n (%)	50 (35.0)	86 (28.2)	0.146
**Individual genotype, n (%)**			
HPV16	84 (58.7)	144 (47.2)	0.023
HPV18	16 (11.2)	15 (4.9)	0.015
HPV31	24 (16.8)	52 (17.0)	0.944
HPV33	8 (5.6)	12 (3.9)	0.428
HPV35	7 (4.9)	10 (3.3)	0.404
HPV39	10 (7.0)	16 (5.2)	0.461
HPV45	5 (3.5)	15 (4.9)	0.497
HPV51	16 (11.2)	23 (7.5)	0.202
HPV52	14 (9.8)	34 (11.1)	0.665
HPV56	4 (2.8)	19 (6.2)	0.125
HPV58	16 (11.2)	27 (8.9)	0.434
HPV59	10 (7.0)	16 (5.2)	0.461
HPV66	7 (4.9)	18 (5.9)	0.665
HPV68	4 (2.8)	13 (4.3)	0.449
**Grouped genotype analysis, n (%)**			
HPV16/18	94 (65.7)	155 (50.8)	0.003
Other nonavalent vaccine genotypes†	57 (39.9)	123 (40.3)	0.925
Other HR-HPV genotypes‡	41 (28.7)	94 (30.8)	0.644

Data are presented as n (%) unless otherwise indicated. Genotype analyses were restricted to HR-HPV-positive cases (2011–2016, n = 143; 2022–2025, n = 305).

Individual genotype percentages are not mutually exclusive because more than one HR-HPV genotype could be detected in the same patient. Multiple HR-HPV infection was defined as the detection of two or more HR-HPV genotypes. Grouped genotype categories were also not mutually exclusive in patients with multiple infections. The HPV16/18 group comprised HPV16 and HPV18; other nonavalent vaccine genotypes comprised HPV31, HPV33, HPV45, HPV52, and HPV58; and other HR-HPV genotypes comprised HPV35, HPV39, HPV51, HPV56, HPV59, HPV66, and HPV68. Abbreviations: HPV, human papillomavirus; HR-HPV, high-risk human papillomavirus; HSIL, high-grade squamous intraepithelial lesion; SD, standard deviation.

Table S2. HR-HPV genotype distribution in 2022–2025 HSIL and invasive SCC

**Genotype**	**HSIL 2022–2025 (n = 305)**	**SCC 2022–2025 (n = 44)**	***p* value**
HPV16	144 (47.2)	29 (65.9)	0.020
HPV18	15 (4.9)	5 (11.4)	0.154
HPV31	52 (17.0)	2 (4.5)	0.032
HPV33	12 (3.9)	4 (9.1)	0.128
HPV35	10 (3.3)	0 (0)	0.621
HPV39	16 (5.2)	1 (2.3)	0.707
HPV45	15 (4.9)	3 (6.8)	0.484
HPV51	23 (7.5)	0 (0)	0.095
HPV52	34 (11.1)	0 (0)	0.013
HPV56	19 (6.2)	3 (6.8)	0.748
HPV58	27 (8.9)	0 (0)	0.034
HPV59	16 (5.2)	1 (2.3)	0.707
HPV66	18 (5.9)	0 (0)	0.144
HPV68	13 (4.3)	1 (2.3)	1.000

Data are presented as n (%). Analyses were restricted to HR-HPV-positive cases. Individual genotype percentages are not mutually exclusive because multiple HR-HPV genotypes could be detected in the same patient. p values were calculated using Pearson's chi-square test or Fisher's exact test when expected cell counts were <5. Abbreviations: HPV, human papillomavirus; HR-HPV, high-risk human papillomavirus; HSIL, high-grade squamous intraepithelial lesion; SCC, squamous cell carcinoma.

Table S3. HR-HPV genotype distribution in 2022–2025 HSIL according to birth cohort

Variable	Born before 1994 (n = 256)	Born in or after 1994 (n = 49)	*p* value
**Individual HR-HPV genotype, n (%)**			
HPV16	127 (49.6)	17 (34.7)	0.055
HPV18	15 (5.9)	0 (0)	0.141
HPV31	45 (17.6)	7 (14.3)	0.574
HPV33	12 (4.7)	0 (0)	0.226
HPV35	7 (2.7)	3 (6.1)	0.206
HPV39	13 (5.1)	3 (6.1)	0.728
HPV45	14 (5.5)	1 (2.0)	0.480
HPV51	18 (7.0)	5 (10.2)	0.390
HPV52	23 (9.0)	11 (22.4)	0.006
HPV56	16 (6.3)	3 (6.1)	1.000
HPV58	16 (6.3)	11 (22.4)	0.001
HPV59	12 (4.7)	4 (8.2)	0.301
HPV66	16 (6.3)	2 (4.1)	0.748
HPV68	10 (3.9)	3 (6.1)	0.446
**Grouped HR-HPV genotypes, n (%)**			
HPV16/18	138 (53.9)	17 (34.7)	0.014
Other nonavalent vaccine genotypes†	95 (37.1)	28 (57.1)	0.009
Other HR-HPV genotypes‡	78 (30.5)	16 (32.7)	0.762

Data are presented as n (%) and analyses were restricted to HR-HPV-positive HSIL cases diagnosed in 2022–2025. Birth cohort was categorized as birth before 1994 or in or after 1994. Individual genotype percentages are not mutually exclusive because multiple HR-HPV genotypes could be detected in the same patient. p values were calculated using Pearson's chi-square test or Fisher's exact test when expected cell counts were <5. Grouped genotype categories are likewise not mutually exclusive in patients with multiple infections. †Other nonavalent vaccine genotypes comprised HPV31, HPV33, HPV45, HPV52, and HPV58. ‡Other HR-HPV genotypes comprised

HPV35, HPV39, HPV51, HPV56, HPV59, HPV66, and HPV68. Abbreviations: HPV, human papillomavirus; HR-HPV, high-risk human papillomavirus; HSIL, high-grade squamous intraepithelial lesion.

Table S4. Genotype-specific frequency of multiple HR-HPV infection according to study cohort

Genotype	HSIL 2011–2016	HSIL 2022–2025	SCC 2022–2025
HPV16	30/84 (35.7)	37/144 (25.7)	3/29 (10.3)
HPV18	8/16 (50.0)	10/15 (66.7)	1/5 (20.0)
HPV31	13/24 (54.2)	25/52 (48.1)	0/2 (0.0)
HPV33	5/8 (62.5)	8/12 (66.7)	0/4 (0.0)
HPV35	6/7 (85.7)	4/10 (40.0)	—
HPV39	7/10 (70.0)	13/16 (81.3)	1/1 (100.0)
HPV45	4/5 (80.0)	9/15 (60.0)	1/3 (33.3)
HPV51	13/16 (81.3)	17/23 (73.9)	—
HPV52	12/14 (85.7)	14/34 (41.2)	—
HPV56	4/4 (100.0)	10/19 (52.6)	2/3 (66.7)
HPV58	11/16 (68.8)	13/27 (48.1)	—
HPV59	9/10 (90.0)	12/16 (75.0)	1/1 (100.0)
HPV66	7/7 (100.0)	11/18 (61.1)	—
HPV68	3/4 (75.0)	12/13 (92.3)	0/1 (0.0)

Data are presented as number of genotype-positive cases with multiple HR-HPV infection/total number of cases positive for the corresponding genotype (%). Multiple HR-HPV infection was defined as detection of at least two HR-HPV genotypes in the same patient. A dash indicates that the corresponding genotype was not detected in that cohort. These analyses are descriptive; percentages for genotypes represented by small numbers of cases should be interpreted cautiously. Abbreviations: HPV, human papillomavirus; HR-HPV, high-risk human papillomavirus; HSIL, high-grade squamous intraepithelial lesion; SCC, squamous cell carcinoma.
